# Persona-Driven Data Augmentation for Disease Name Recognition Across Rare and General Disease Corpora: Comparative Evaluation Study

**DOI:** 10.2196/87831

**Published:** 2026-07-24

**Authors:** Jude Crener Junior Pierre, Tomohiro Nishiyama, Shaowen Peng, Shoko Wakamiya, Eiji Aramaki

**Affiliations:** 1 Nara Institute of Science and Technology Ikoma, Nara Japan

**Keywords:** data augmentation, disease, large language model, LLM, low-resource, named entity recognition, natural language processing, NER, NLP, persona, rare disease

## Abstract

**Background:**

Medical information extraction requires automatically identifying disease names and related terms in text. This task, known as named entity recognition (NER), relies on expert-annotated data that are costly to produce and often available only in limited quantities. Data augmentation (DA) aims to expand available training data; however, standard techniques such as synonym replacement and back-translation may introduce inappropriate substitutions or fail to preserve entity-label alignment, which is critical for sequence-labeling tasks. Although large language models can generate fluent text, their outputs may also contain factual inconsistencies or unintended changes if not carefully controlled.

**Objective:**

This study investigated whether persona-driven, document-level DA using a large language model could improve biomedical disease NER performance by generating diverse rephrasings of medical documents while preserving annotated entities.

**Methods:**

We designed a DA framework using multiple personas that varied in medical expertise, personality, tone, and narrative style. Using prompting constrained by XML tags, each persona rephrased training documents while aiming to preserve annotated entity spans. We evaluated the framework on 2 biomedical disease NER datasets with complementary roles: RareDis, a low-resource rare disease corpus, and National Center for Biotechnology Information (NCBI) disease, a more general disease benchmark. Semantic fidelity and lexical diversity were measured using BERTScore and Bilingual Evaluation Understudy (BLEU-4), respectively, and personas were grouped into high-, balanced-, and low-fidelity subsets. Biomedical pretrained BioBERT models were fine-tuned and evaluated under multiple settings, including gold-standard (GS) data only, synonym replacement, single-persona augmentation, curated persona subsets, and all-persona augmentation. Performance was assessed using microaveraged entity-level precision, recall, and *F*_1_-score, and results were examined at both the overall and individual entity-type levels. Performance values are reported as mean (SD).

**Results:**

Persona-driven DA improved NER performance over GS-only training in both datasets, with the strongest gains obtained by combining multiple persona-generated variants with GS data. In RareDis, the best result was achieved by the low-fidelity subset (mean *F*_1_-score 73.35, SD 0.19 vs baseline 71.22, SD 0.45), while in NCBI disease, the all-personas setting performed best (mean *F*_1_-score 89.32, SD 0.26 vs baseline 87.82, SD 0.18). In low-resource experiments, the all-personas and high-fidelity persona settings in NCBI disease exceeded the performance of the model trained on 100% GS data using only 60% of the training data, whereas gains in RareDis were more modest. Entity-level analysis showed improvements across RareDis categories, particularly for symptom, and confusion analysis indicated reduced symptom-sign confusion under augmentation.

**Conclusions:**

Persona-driven DA improved biomedical disease NER by introducing controlled linguistic variation while largely preserving annotated entities. The strongest gains were obtained when multiple persona-generated variants were combined with GS data, although the benefit varied across datasets. These findings suggest that this approach is a promising strategy for low-resource biomedical NER.

## Introduction

The identification of medical conditions in clinical texts and the literature relies on natural language processing (NLP) techniques, particularly named entity recognition (NER), which detects words or token spans and categorizes them into established medical categories [1]. In the biomedical domain, this task is also known as biomedical NER (BioNER) and typically focuses on entities such as diseases, drugs, and proteins. Accurate NER supports many medical applications. It enables the creation of structured information from electronic health records by extracting entities from unstructured notes, supports pharmacovigilance by detecting adverse drug events in clinical text, and supports precision medicine through patient phenotyping [2-7].

NER has long been recognized as a cornerstone of information extraction, with its importance first highlighted during the Message Understanding Conference-6 (MUC-6) [8]. Early NER systems relied on rule-based and dictionary-driven methods, which provided modest accuracy but were limited in scalability [9,10]. These were gradually replaced by statistical models [11-14] and later by deep learning approaches that incorporated transformer-based architectures such as BERT (Bidirectional Encoder Representations from Transformers) [15-17]. More recently, the introduction of large language models (LLMs) has further advanced the scalability of NER tasks [4,5].

Despite these benefits, progress in BioNER remains limited by the scarcity of annotated data. Existing datasets are often small, unbalanced, and restricted to narrow domains, which can lead to model overfitting and weaken generalization. This low-resource setting represents a major barrier across medical NLP tasks, since annotation requires costly domain expertise [18,19].

Data augmentation (DA) has emerged as a strategy to address this challenge by generating synthetic examples from existing data. Traditional methods such as back-translation, synonym replacement, and rule-based perturbations have shown promising results for tasks involving single sentences (eg, sentiment analysis and text classification) and sentence-pair tasks (eg, natural language inference and machine translation), as long as sentence coherence is preserved [20-23]. However, for NER, these approaches are often less effective: they may fail to maintain entity–label consistency, struggle to produce diverse yet coherent outputs, and perform poorly with specialized biomedical terminology [21,24,25]. In particular, BioNER DA presents additional challenges, as many existing methods rely on external linguistic resources, such as dependency parsers and WordNet, which are designed for the general domain rather than biomedical text. As a result, they often produce incorrect parses and inappropriate substitutions. Other strategies depend on training task-specific language models, which increases computational costs and limits applicability in low-resource settings [24]. Together, these limitations motivate the evaluation of more controllable augmentation strategies for BioNER.

The recent rise of generative AI systems with LLMs has created new opportunities. These models can generate fluent, natural-sounding text for biomedical DA. However, they also introduce risks, such as factual inaccuracies, limited lexical diversity, and scientific inconsistencies that can degrade model performance if used directly without supervision as training data. To address these issues, careful prompt design and postprocessing are necessary to ensure quality and accuracy [19,26].

In this study, we introduce a controlled, document-level DA framework for BioNER based on persona-conditioned LLM prompting. Instead of traditional sentence-level paraphrasing, our method generates document-level rephrasings using persona-conditioned prompts that introduce controlled linguistic variation through differences in medical expertise, communication style, and narrative perspective. By constraining entity mentions within XML tags, the framework is intended to preserve annotated spans while allowing controlled variation in the surrounding nonentity text.

To evaluate the proposed method across biomedical disease NER settings, we assess it on 2 datasets with complementary roles: a low-resource rare disease corpus and a more general disease-focused benchmark. Rare disease NER serves as an important motivating case for low-resource biomedical information extraction because such conditions are associated with limited clinical exposure, specialized terminology, and costly expert annotation [27-32]. In contrast, the more general disease benchmark allows us to examine whether the effects of persona-driven DA are specific to the rare disease setting or also hold in a broader disease NER context. Together, these datasets enable a more comprehensive evaluation across specialized and more general biomedical disease NER conditions.

Specifically, we address the following 3 research questions (RQs):

- RQ1: can persona-driven DA improve disease NER performance over nonaugmented training?
- RQ2: which persona types are most effective for generating useful rephrasings?
- RQ3: how do persona selection strategies (all vs curated subsets) influence NER performance?

Through these questions, we examine the utility of persona-driven DA for biomedical disease NER, including its effects on performance, linguistic variation, and the relative value of curated subsets vs all-persona combinations. By studying these factors across complementary disease NER settings, this study provides a broader evaluation of performance and linguistic variation.

## Methods

### Datasets

We evaluated the proposed augmentation framework on 2 biomedical disease NER datasets: RareDis and National Center for Biotechnology Information (NCBI) disease. RareDis served as the primary low-resource rare disease benchmark, whereas NCBI disease was included as an additional disease-focused corpus for broader validation beyond the rare disease setting.

RareDis is a corpus specifically developed for rare disease NER and was built from 1041 English texts collected from the National Organization for Rare Disorders (NORD) website [33]. RareDis contains 4 main entity types: rare disease, disease, sign, and symptom. Diseases represent general medical conditions commonly encountered in clinical settings. Rare diseases, by contrast, are low-prevalence conditions formally recognized as distinct clinical entities. Signs refer to objective findings observed during physical examination or laboratory testing, whereas symptoms denote subjective experiences reported by patients that cannot be directly observed through medical tests. We used the original split of 729 training documents, 104 development documents, and 208 test documents.

NCBI disease is a biomedical disease corpus of 793 documents derived from PubMed abstracts [34]. Compared with RareDis, which consists of rare disease descriptions, NCBI disease reflects scientific biomedical literature that is typically more concise and terminologically dense. We used the standard split of 593 training documents, 100 development documents, and 100 test documents.

The 2 datasets also differ in annotation scope. RareDis includes 4 clinically related entity categories, making it suitable for analyzing confusion among semantically close labels, whereas NCBI disease focuses on disease mentions only. Together, these datasets allowed us to assess the proposed method in both a multiclass rare disease NER setting and a broader disease-focused BioNER setting.

### Persona Design

**Figure 1:**
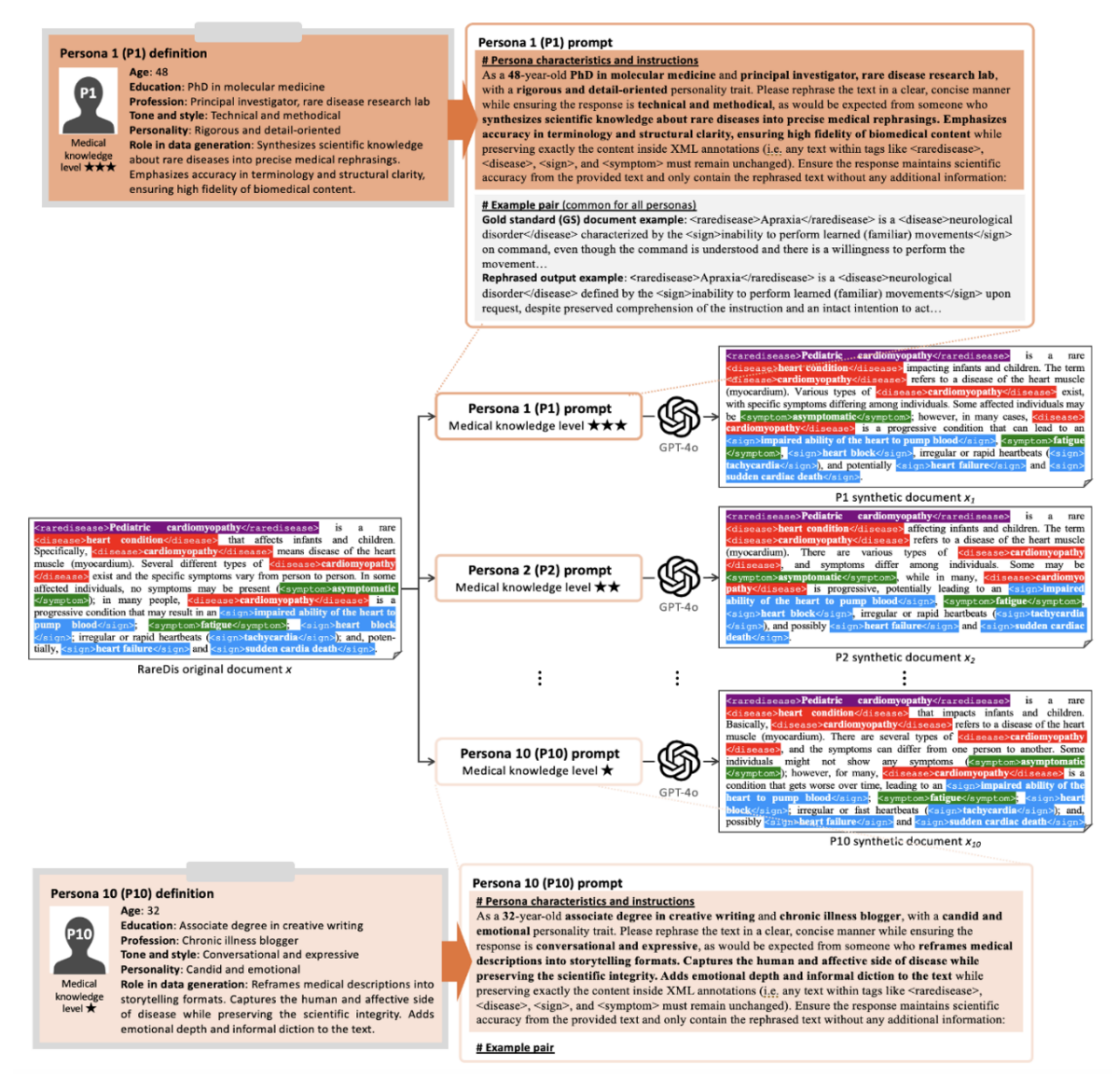
Persona-driven document-level data augmentation (DA) pipeline. Original documents with XML-tagged biomedical entities are rephrased by the LLM (GPT-4o) following persona-conditioned prompts. Persona definitions guided linguistic variation and helped ensure that gold-standard entity boundaries were preserved. Examples of synthetic outputs (P1, P2, and P10) illustrate controlled variation across medical knowledge levels.

To introduce controlled stylistic variation during the document rephrasing process in the proposed DA method, we introduced a persona-driven data generation framework, as shown in Figure 1. We defined 10 personas with distinct characteristics, each representing a different level of medical expertise, from expert clinicians to nonspecialist narrators, while differing in age, educational background, and professional focus. In addition, personas were distinguished by different characteristics such as tone, writing style, and personality traits, which influenced the formality and narrative perspective of the generated outputs. Each persona also included a role-specific description that provided additional instructions for the generative model, guiding the LLM to produce rephrasings that consistently reflected the intended persona. The same persona set was applied across both RareDis and NCBI disease to support comparable evaluation across datasets. Complete persona definitions are reported in Table 1.

**Table 1:** Characteristics of the 10 personas used to guide synthetic text generation. For each persona, we report demographic background, communication style, personality traits, and the specific role intended to shape the generated medical narratives.

Persona ID, age (years), and education	Profession	Tone and style	Personality traits	Role in data generation
P1; 48; PhD in molecular medicine	Principal investigator: rare disease research laboratory	Technical and methodical	Rigorous and detail-oriented	Synthesizes scientific knowledge about rare diseases into precise medical rephrasings. Emphasizes accuracy in terminology and structural clarity, ensuring high fidelity of biomedical content.
P2; 50; MD (cardiology)	Urban cardiologist	Analytical and detailed	Precise and innovative	Conveys complex and technical insights into precise, structured explanations, ensuring that intricate medical information is communicated in an accessible and clinically relevant manner.
P3; 38; PharmD	Clinical Pharmacist	Concise and pragmatic	Detail-oriented and cautious	Translates clinical-medication interactions and disease mechanisms into practical, structured explanations. Focuses on treatment implications and physiological clarity, avoiding overgeneralizations while maintaining clinical accuracy.
P4; 45; nursing	Community family physician	Warm and accessible	Compassionate and pragmatic	Synthesizes advanced clinical insights into accessible explanations, effectively bridging expert-level knowledge and everyday language for a broader audience.
P5; 41; master’s in science communication	Health journalist	Neutral and explanatory	Curious and balanced	Explains complex health topics for a general audience using journalistic clarity. Bridges scientific accuracy with accessible narrative flow, offering well-informed yet nontechnical language aligned with public health communication norms.
P6; 36; BEd in biology education	High school biology teacher	Clear and analogy-driven	Patient and enthusiastic	Uses pedagogical framing and familiar metaphors to explain difficult medical ideas. Focuses on simplicity, using teaching logic to convey meaning.
P7; 35; bachelor’s in science communications	Patient advocate	Empathetic and engaging	Approachable and articulate	Communicates health information in an accessible and motivating way, translating clinical data into actionable advice for patients.
P8; 58; high school certificate	Family caregiver (elderly mother)	Gentle and anecdotal	Empathetic and observant	Rephrases medical narratives through lived caregiving experience, often focusing on symptoms and outcomes. Prioritizes clarity, emotional tone, and lay understanding, especially around patient experience and interpretation.
P9; 40; high school diploma	Informed layperson	Conversational and straightforward	Pragmatic and resourceful	Represents the everyday perspective, highlighting common health challenges, concerns, and lay interpretations of medical information.
P10; 32; associate degree in creative writing	Chronic illness blogger	Conversational and expressive	Candid and emotional	Reframes medical descriptions into storytelling formats. Captures the human and affective side of disease while preserving the scientific integrity. Adds emotional depth and informal diction to the text.

### Synthetic Data Generation Pipeline

For synthetic data generation, only documents in the training split were augmented. Each document in the RareDis training set and the NCBI disease training set was rephrased once per persona, resulting in 10 synthetic variants per source document. Throughout the text and figures, we refer to the original training documents from both datasets as gold-standard (GS) and to persona-generated variants as Pn (n = 1 – 10).

The process was implemented using the GPT-4o (OpenAI) generative model with persona-customized prompts in a one-shot format. Each prompt specified the persona’s characteristics and instructions and included a single example of a GS document paired with its rephrased version to illustrate the desired transformation style. The illustrative example was first generated in a zero-shot setting and manually reviewed to confirm that XML-tagged entities were preserved and that the output matched the intended persona profile. The same single RareDis training document was used as the illustrative example across all personas and for both datasets to keep the prompting setup consistent; this example also included disease mentions, making it compatible with the NCBI disease setting. For GPT-4o text generation, the temperature parameter was set to 0 to reduce output variability and encourage more consistent, controlled rephrasings across personas.

To preserve annotation quality, all prompts explicitly instructed the model to keep entity mentions wrapped in XML tags unchanged. This constrained rephrasing to nonentity spans and helped ensure that GS entity boundaries were preserved. A descriptive statistical overview of the persona-based variants and GS documents across both datasets is provided in Tables S1 and S2 in Multimedia Appendix 1.

### Baselines and Comparison Methods

To evaluate the contribution of persona-driven DA, we compared the proposed method with 2 baseline settings: a GS baseline and a synonym replacement (SR) baseline. In the GS setting, models were trained using only the original GS training data without augmentation. This served as the primary reference condition for both RareDis and NCBI disease.

As a traditional augmentation baseline, we used SR following Dai and Adel [25]. We used the authors’ released implementation from the associated GitHub repository [35] to generate the SR-augmented training data. In this NER-oriented formulation, selected tokens are replaced with WordNet synonyms, and labels are updated when multitoken synonyms are introduced, meaning that the original label sequence is not always preserved. Similar to persona-driven DA, SR was applied only to the training splits. However, unlike the proposed XML-constrained persona-driven framework, SR does not explicitly preserve original entity mentions. SR therefore served as a conventional lexical-perturbation baseline for comparison with the proposed method.

In addition to these baselines, we evaluated several persona-driven DA settings in which the same original GS training data were augmented using either a single persona, selected persona subsets, or all personas. Subsets were defined either through development-set selection or by grouping personas according to their fidelity/diversity characteristics. These settings were used to examine whether the effectiveness of the proposed framework depended primarily on individual personas, selected persona combinations, or broader stylistic diversity.

### Evaluation Metrics

To assess the quality of the generated texts, we used 2 complementary metrics. Semantic fidelity was measured using BERTScore [36], which compares reference and candidate texts using contextual token embeddings rather than exact word matches. Given a reference text *x* and a candidate text *x̂*, BERTScore precision, recall, and *F*_1_-score are defined as:

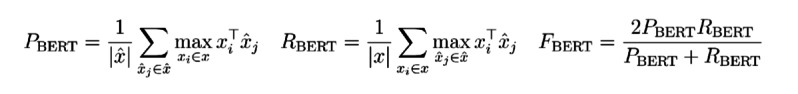



Where *x*ᵢᵀ *x̂*ⱼ denotes the cosine similarity between contextual token embeddings. Higher BERTScore indicates stronger semantic preservation of the GS text.

Lexical variation was characterized using Bilingual Evaluation Understudy (BLEU) [37], specifically BLEU-4, which measures modified n-gram overlap up to 4-grams. BLEU-4 is computed as:

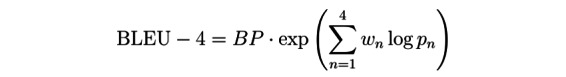



where *p_n_* is the modified n-gram precision, *BP* is the brevity penalty, and *w_n_*=1/4. In our setting, lower BLEU-4 indicates greater surface variation from the GS text, whereas higher BLEU-4 indicates closer lexical overlap. For each GS training document in each dataset, we compared it with its persona-driven rephrased version and averaged the resulting BERTScore and BLEU-4 values across outputs for each persona.

For downstream NER evaluation, we used microaveraged entity-level precision, recall, and *F*_1_-score with strict span matching. Using the *seqeval* library, a predicted entity was considered correct, or a true positive (TP), only if its boundary and entity type exactly matched those of a GS entity. Predictions with incorrect boundaries or labels, as well as additional predicted entities not present in the GS, were counted as false positives (FPs), whereas GS entities missed by the model were counted as false negatives (FNs). Formally,

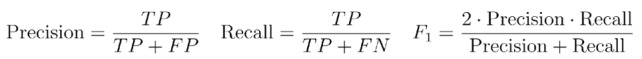



### Experimental Setting

All models were trained and evaluated separately on RareDis and NCBI disease using their respective standard training, development, and test splits. We adopted the standard inside-outside-beginning tagging scheme for NER, in which B- marks the beginning of an entity span, I- marks its continuation, and O marks all nonentity tokens. For the model backbone, we used BioBERT (dmis-lab/biobert-v1.1) [17], a BERT [16] model pretrained on biomedical texts.

Training and evaluation were conducted at the sentence level. Each document was first split into sentences. XML tags were removed, and the sentences were tokenized so that input tokens could be paired with their corresponding entity labels. During training, these token-label pairs were fed into the model to optimize entity predictions. During evaluation, sentences from the test set were tokenized in the same way but without labels, allowing the model to output predicted entity tags for comparison with the GS test set.

Models were trained with a maximum sequence length of 256 tokens, a batch size of 32, a learning rate of 1e-5, and the AdamW optimizer. Training was run for up to 10 epochs, with early stopping applied if no improvement was observed on the development set for 5 consecutive epochs. Each experiment was repeated 3 times using different seeds. For each run, the checkpoint with the best development-set *F*_1_-score was retained for final evaluation. Final precision, recall, and *F*_1_-score were then summarized across the 3 runs and reported as mean values with their corresponding SDs. All experiments were conducted on a single NVIDIA RTX PRO 6000 graphics processing unit.

### Ethical Considerations

This study used previously collected BioNER datasets. The original dataset publications describe the data sources and collection procedures. NCBI disease is publicly available, and RareDis was used in accordance with the access conditions specified by its original authors. According to the Ethical Guidelines for Medical and Health Research Involving Human Subjects in Japan, this study does not fall under research involving human subjects and is not subject to ethical review. This determination is consistent with the institutional regulations of Nara Institute of Science and Technology [38]. Because this study involved only secondary analysis of existing datasets and did not involve new data collection from human participants, identifiable personal information, identifiable participant images, or participant compensation, formal ethics review and additional informed consent were not required.

## Results

### Overview

Using our persona-driven DA framework, we generated multiple rephrasings of the RareDis and NCBI disease training documents and evaluated their impact on NER performance. The use of distinct personas introduced measurable and meaningful linguistic variation. Our evaluation covered several stages: analysis of the fidelity and diversity of generated texts, evaluation of individual persona settings, comparison of augmentation settings that combined persona-generated texts with GS data, low-resource experiments, and error analysis.

### Diversity vs Fidelity Landscape

**Figure 2:**
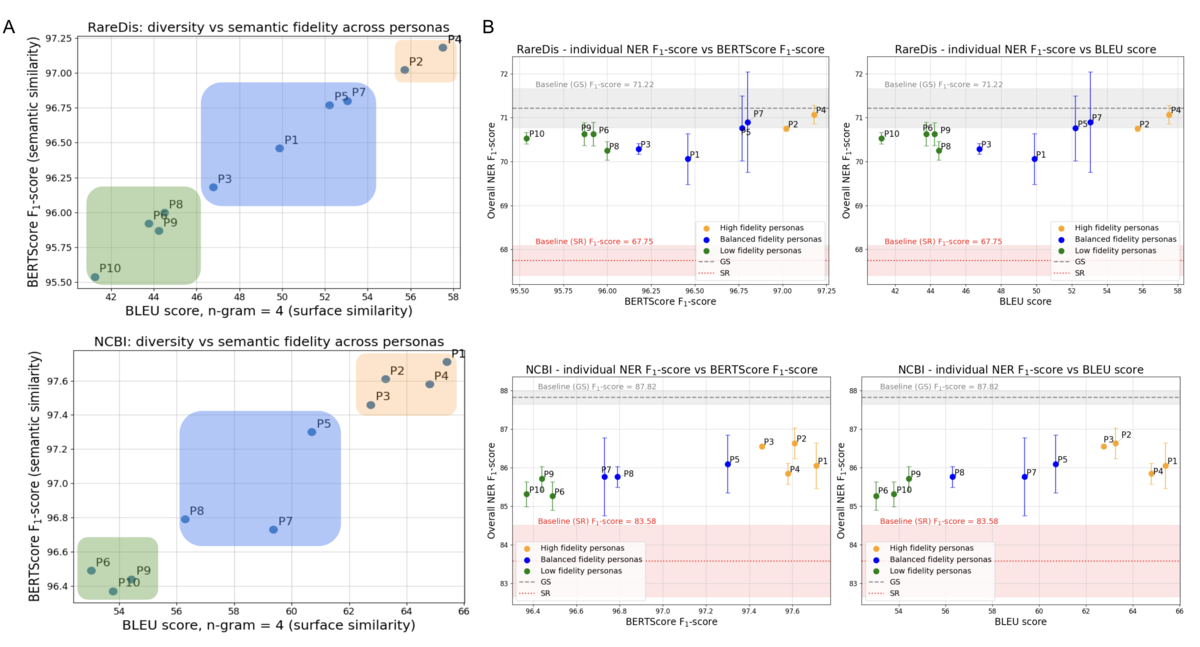
Diversity–fidelity landscape and its relation to named entity recognition (NER) performance across personas in the RareDis and National Center for Biotechnology Information (NCBI) disease datasets. (A) Plots persona-generated texts by BERTScore F1-score and Bilingual Evaluation Understudy-4 (BLEU-4) for RareDis and NCBI disease, respectively, where BERTScore F1-score reflects semantic fidelity to the gold-standard (GS) texts and lower BLEU-4 indicates greater lexical diversity. Shaded regions denote high-, balanced-, and low-fidelity persona groups. (B) NER F1-score for each persona setting in relation to BERTScore F1-score and BLEU-4. Dashed and dotted horizontal lines indicate the GS-only and synonym replacement (SR) baselines.

We first assessed the quality of the persona-generated texts by quantifying both semantic fidelity and lexical diversity, as shown in Figure 2A and Tables S3 and S4 in Multimedia Appendix 1. Fidelity refers to how well the rephrased text preserves the semantic content of the GS texts, which is important for avoiding unintended changes in the training data. Diversity, on the other hand, measures the degree of surface-level variation in wording and phrasing, which may help reduce overfitting, expose the model to alternative lexical forms, and support generalization to unseen texts.

BERTScore evaluated semantic fidelity by comparing persona-generated texts against the GS texts, while BLEU-4 was used to assess surface-level similarity and relative variation across persona outputs. Overall, both datasets showed 3 broad persona groupings. In RareDis, high-fidelity personas (P2 and P4) achieved the highest BERTScore (≥97.0) and BLEU-4 (≥55), generating outputs that remained very close to the GS texts with limited stylistic variation. Low-fidelity personas (P6, P8, P9, and P10) showed the lowest values (BERTScore ≤96.0; BLEU-4 ≤45), indicating the most diverse but least faithful outputs. Balanced-fidelity personas (P1, P3, P5, and P7) occupied the middle range (BERTScore: 96.1-96.8; BLEU-4: 46-53). In NCBI disease, high-fidelity personas (P1, P2, P3, and P4) also formed the top cluster (BERTScore ≥97.4; BLEU-4 ≥62.0), whereas low-fidelity personas (P6, P9, and P10) showed the lowest values (BERTScore ≤96.5; BLEU-4 ≤54.5). Balanced-fidelity personas (P5, P7, and P8) remained in the intermediate range (BERTScore 96.7–97.3; BLEU-4 56–61).

Overall, NCBI disease showed generally higher BERTScore and BLEU-4 values than RareDis, indicating that persona-generated texts remained closer to the GS texts. Although several personas remained relatively strong across datasets, the overall clustering pattern was not identical, indicating that fidelity and diversity associated with each persona depended in part on the linguistic properties of the target corpus.

### Individual Persona NER Performance

We next evaluated the effect of training solely on persona-generated texts, without combining them with the GS data. In this setting, each model trained on a single persona’s outputs was evaluated on the RareDis and NCBI disease test sets (Figure 2B). The results suggested that individual-persona NER performance tended to align more closely with higher-fidelity outputs than with more lexically diverse, lower-fidelity outputs. In RareDis, P4 and P7 achieved the highest average *F*_1_-scores, with P4 performing best. These results included both high-fidelity and balanced-fidelity personas, with other personas such as P2 and P5 also remaining competitive. In NCBI disease, high-fidelity personas again tended to perform best, especially P2 and P3, although the ranking pattern was not identical across datasets. By contrast, low-fidelity personas, which introduced greater lexical variation, generally produced lower *F*_1_-scores.

These results reveal an important property of the framework: no single persona captures sufficient linguistic diversity to match the GS training data on its own, while all individual persona settings consistently remained above the SR baseline, confirming that persona-generated texts preserve task-relevant information. This pattern suggests that the strength of persona-driven DA lies not in any individual persona but in the complementary diversity that emerges when multiple perspectives are combined, with each persona contributing a distinct stylistic dimension that no single rephrasing style can replicate alone. Consequently, we next examined whether combining persona-generated texts with GS texts offered a more effective strategy for improving NER performance.

### Comparison of Persona-Mix Augmentation Strategies

We then examined whether combining persona-generated texts could lead to stronger improvements in NER performance. In the single-persona DA setting (GS + P), each persona’s outputs were added to the GS texts. In the curated-subset DA setting, we tested combinations based on the fidelity clusters, as well as a top-3 subset formed from the strongest individual personas based on development-set performance in the single-persona (GS + P) setting, as shown in Table S5 in Multimedia Appendix 1. The top-3 personas were P2, P4, and P7 for RareDis, and P2, P3, and P8 for NCBI disease. Detailed test-set precision, recall, and *F*_1_-scores, together with their SDs, are provided in Tables S6 and S7 in Multimedia Appendix 1.

**Figure 3:**
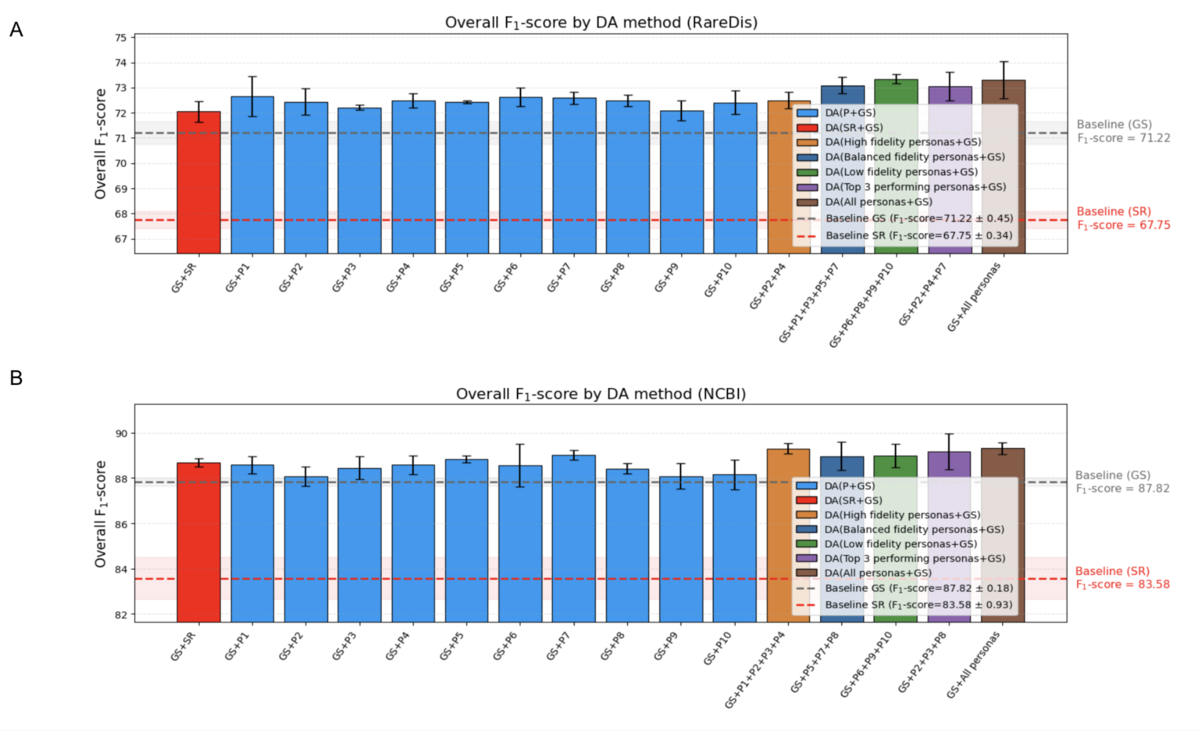
Overall F1-scores for single-persona and curated-subset data augmentation (DA) settings on the (A) RareDis and (B) National Center for Biotechnology Information (NCBI) disease datasets. The dashed horizontal lines indicate the gold-standard (GS) and synonym replacement (SR) baseline performances.

As shown in Figure 3, combining personas was generally more effective than relying on a single persona. In RareDis, the low-fidelity subset achieved the best overall performance, followed closely by the all-personas setting, while the balanced-fidelity and top-3 subsets also produced strong gains. In NCBI disease, the all-personas setting achieved the highest mean *F*_1_-score, followed closely by the high-fidelity and top-3 subsets.

This trend was also reflected in the per-entity analysis presented in Tables S8 and S9 in Multimedia Appendix 1. In RareDis, the best scores were obtained by broader persona mixtures rather than by any single-persona DA setting, with the top-3 subset performing best for disease, the all-personas setting for rare disease, and the low-fidelity subset for sign and symptom. In NCBI disease, the highest disease *F*_1_-score was achieved by the all-personas setting. Although differences in entity counts across augmentation settings (Tables S1 and S2 in Multimedia Appendix 1) may have contributed in part to some of these patterns, they do not fully account for them.

### Low-Resource NER

**Figure 4:**
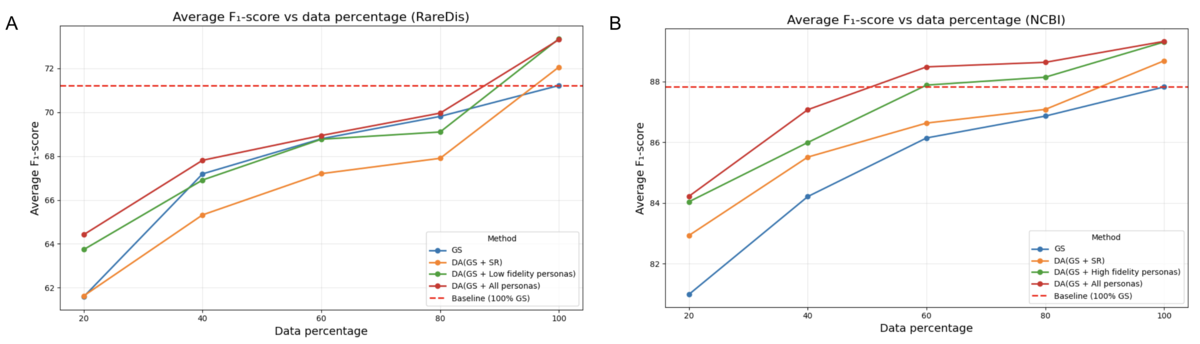
Average F1-scores across training data percentages (20%-100%) for gold-standard (GS) and selected augmentation settings on (A) RareDis and (B) National Center for Biotechnology Information (NCBI) disease. The dashed horizontal line indicates the baseline performance obtained with 100% GS data.

Because manually annotated biomedical corpora are often limited, we further examined whether persona-based augmentation could improve data efficiency under low-resource conditions. To do so, we randomly subsampled 20% to 100% of the GS training data, in 20% increments, together with the corresponding augmented data. For each seed, document subsampling was performed independently at each training percentage, and results were averaged across the 3 runs. We then compared 4 settings: GS-only, GS + SR, GS combined with the best-performing curated subset for each dataset (low-fidelity for RareDis and high-fidelity for NCBI disease), and GS combined with all personas (Figures 4A and 4B). The results showed that the gains from augmentation were larger in NCBI disease than in RareDis. Detailed low-resource average *F*_1_-scores and SDs per training data percentage are provided in Tables S13 and S14 in Multimedia Appendix 1.

In NCBI disease, GS combined with all personas achieved the highest average *F*_1_-scores across training-data percentages, while the high-fidelity subset also performed strongly. Both settings surpassed the 100% GS baseline using only 60% of the GS training data. In RareDis, the all-personas setting performed best from 20% to 80% of the GS training data, whereas the low-fidelity subset achieved the highest score under the 100% training condition. However, the gains were modest, and no augmented setting surpassed the 100% GS baseline before the full training condition. This likely reflects the greater difficulty of RareDis as a multiclass corpus with specialized terminology and fine-grained entity distinctions. Overall, these findings indicate that persona-driven DA produced clearer gains for NCBI disease under low-resource settings, while its effects on RareDis were more moderate.

### Error Analysis of Generated Entities and NER Confusions

Although XML-constrained prompting was designed to preserve GS entity spans, analysis of the generated texts revealed occasional modifications by the LLM. To identify candidate cases, we compared XML-annotated entities of the same label between the GS and persona-generated texts using Python’s built-in library *difflib.SequenceMatcher*. Cases with a similarity score of 1.0 were treated as unchanged and excluded, whereas cases with scores between 0.70 and 0.99 were treated as modified entities. Representative examples are shown in Table 2.

**Table 2:** Examples of entity-level modifications in persona outputs. The table compares gold-standard (GS) and persona-generated sentences, highlighting cases in which entities were modified.

Source	Gold-standard text	Persona-generated text
RareDis	...is caused by a <sign>*deficiency of an enzyme galactose-1-phosphate uridylyl transferase*</sign> (GALT)...	...results from a <sign>*deficiency of the enzyme galactose-1-phosphate uridylyl transferase*</sign> (GALT)...
RareDis	...This causes the <sign>*eyes to become red*</sign> and may cause <sign>blurred vision</sign>...	...leading to <sign>*redness of the eyes*</sign> and potentially <sign>blurred vision</sign>...
RareDis	...is a rare <disease>*cancer of the nasal cavity and/or paranasal sinuses*</disease>...	...is a rare form of <disease>*cancer affecting the nasal cavity and/or paranasal sinuses*</disease>...
NCBI^a^	...confirming the <disease>*Pelizaeus Merzbacher disease*</disease>, but neither the aunt nor the fetus carried a duplication.	...confirming the <disease>*Pelizaeus-Merzbacher disease*</disease> diagnosis, but neither the aunt nor the fetus had the duplication.
NCBI	...indicated no close linkage between the <disease>*CM deficienty*</disease> and the HLA system...	...showed no close linkage between the <disease>*CM deficiency*</disease> and the HLA system...

^a^NCBI: National Center for Biotechnology Information.

The selected examples in Table 2 indicate that these changes were generally minor lexical or orthographic reformulations, such as rewording sign expressions, adding function words, or slightly reformulating disease mentions, while usually preserving the underlying medical meaning. Overall, entity integrity was strongly preserved across both datasets: entity modification rates ranged from 0.79% (72/9134) to 1.60% (146/9134) in RareDis (Table S11 in Multimedia Appendix 1) and from 0.21% (11/5246) to 0.42% (22/5246) in NCBI disease (Table S12 in Multimedia Appendix 1), confirming that XML-constrained prompting was highly effective at preventing unintended entity changes.

As also reflected in Tables S1 and S2 in Multimedia Appendix 1, persona-generated texts sometimes contained slightly fewer entity occurrences than the GS texts, likely because some personas did not always preserve the same frequency of repeated entity mentions when an entity appeared multiple times in the original document. For training, token-label pairs were generated from the final XML annotations in each synthetic document. Therefore, omitted repeated mentions simply did not appear as labeled spans, while tagged, reformulated mentions remained aligned with their labels.

**Figure 5:**
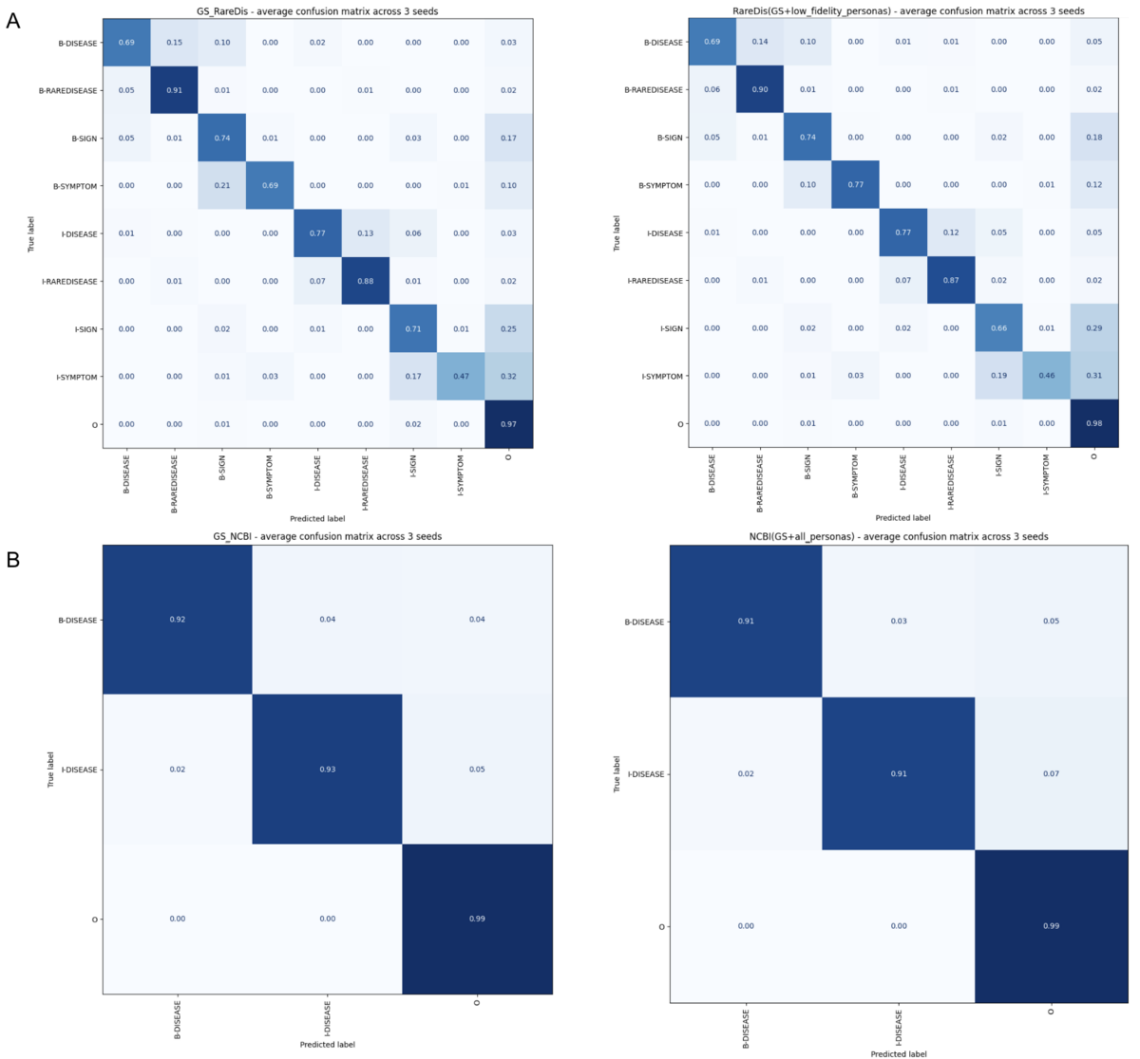
Average normalized confusion matrices across 3 seeds for the gold-standard (GS) baseline and the best-performing augmented model on (A) RareDis and (B) National Center for Biotechnology Information (NCBI) disease.

Beyond entity preservation, we analyzed model errors using normalized confusion matrices averaged across 3 seeds for the GS baseline and the best-performing augmented method in each dataset (Figures 5A and 5B). In RareDis, both models showed recurrent confusions between semantically close classes, especially disease vs rare disease and symptom vs sign. A visible improvement from augmentation was a reduction in symptom-sign confusion, whereas disease-rare disease confusions remained largely similar overall. In NCBI disease, confusion patterns were simpler, and augmentation produced only minor changes. Qualitative examples in Table S10 in Multimedia Appendix 1 show that augmentation corrected some baseline errors, although some confusions remained.

## Discussion

### Overview

In this study, we introduced a persona-driven, document-level DA framework for biomedical disease NER and evaluated it on both a rare disease corpus and a more general disease benchmark. Our findings indicate that persona-driven DA can be applied in a way that largely preserves annotated entity spans and can improve baseline performance, particularly when multiple persona-generated variants are combined with the original training data. More broadly, the results provide insight into which persona types are most useful, how persona selection strategies influences performance, and how this framework may support low-resource training.

### RQ1: Can Persona-Driven DA Improve Disease NER Performance Over Nonaugmented Training?

Our experiments show that persona-driven DA can improve disease NER performance over nonaugmented training, although the benefit depended on the augmentation strategy and the target corpus. In both datasets, the strongest gains came from combining multiple persona-generated variants with the GS data rather than using any single persona alone. In RareDis, the low-fidelity subset performed best, followed closely by the all-personas setting, whereas in NCBI disease, the all-personas setting achieved the highest mean *F*_1_-score, with the high-fidelity and top-3 subsets also performing strongly. The low-resource analysis further supported this pattern. In NCBI disease, the all-personas and high-fidelity persona settings surpassed the baseline trained on 100% of the GS data using only 60% of the training data, whereas in RareDis, the gains were more modest and no augmented setting exceeded the full-data GS baseline until the 100% training condition. Taken together, these findings suggest that persona-driven DA can improve performance and, in some cases, data efficiency, although its impact depends on the characteristics of the target corpus.

### RQ2: Which Persona Types Are Most Effective for Generating Useful Rephrasings?

Our results suggest that high-fidelity personas were the most effective when used individually. This pattern may reflect a more favorable balance between semantic fidelity and useful linguistic variation during rephrasing. In RareDis, P4 achieved the strongest individual performance, while in NCBI disease, the best results came from P2 and P3. By contrast, low-fidelity personas, although more diverse, generally performed less well when used alone. However, when combined with the GS data, personas from different fidelity groups contributed complementary strengths, suggesting that effective rephrasings depend on balancing semantic preservation with controlled linguistic variation.

### RQ3: How Do Persona Selection Strategies Influence NER Performance?

Our comparison of augmentation strategies showed that the most effective persona selection strategy differed across datasets, indicating that no single strategy was uniformly optimal. This difference was also evident in the dataset-specific top-3 curated subsets selected from development-set performance in the GS + P setting: P2, P4, and P7 for RareDis, and P2, P3, and P8 for NCBI disease. Notably, these curated subsets included personas from different fidelity groups, suggesting that combining different rephrasing styles may be beneficial. Broader persona mixtures also introduced more synthetic training examples, so the observed gains may reflect both increased stylistic diversity and increased data volume. Overall, these findings indicate that persona selection matters, but the strongest strategy depends on the target corpus.

### Practical Implications of Persona-Driven Augmentation

The present findings suggest that persona-driven DA may be particularly useful in BioNER settings in which annotated data are limited. In such contexts, generating controlled rephrasings of existing documents may help expand linguistic coverage without requiring additional manual annotations. The observed gains were not uniform across datasets, indicating that the practical benefit of this approach is likely to depend on corpus characteristics and task conditions.

More broadly, the proposed framework shows that it is possible to generate multiple rephrased versions of biomedical texts while largely preserving annotated entity spans. This may be useful for other BioNER settings that face similar challenges related to limited annotated data and specialized terminology.

### Limitations and Future Work

While promising, this work has several limitations. Although we evaluated the proposed framework on 2 biomedical disease NER datasets, RareDis and NCBI disease, both are disease-focused benchmarks. Further validation on other biomedical entity types, clinical text settings, and additional domains is needed to establish the broader generalizability of persona-driven DA.

Another limitation concerns the design of the personas. Their characteristics were selected heuristically based on intuition about what might produce useful variation in biomedical rephrasings. Although the results suggest that this strategy was effective, it was not derived from a systematic optimization procedure and may not transfer equally well to other tasks or corpora.

A further limitation is that we did not evaluate all possible persona combinations. With 10 personas, the combinatorial space is large, and exhaustive evaluation would be computationally costly. Instead, we focused on representative settings, including individual personas, fidelity-based subsets, top-3 curated subsets, and the all-personas configuration, in order to capture the main contrasts relevant to our research questions.

Although the prompting framework was designed to preserve XML-tagged entities, quantitative analysis confirmed that modifications were rare, with rates remaining below 2% in RareDis and below 0.5% in NCBI disease (Tables S11 and S12 in Multimedia Appendix 1). Where modifications did occur, they were minor lexical or orthographic reformulations that generally did not alter the underlying medical meaning. Some persona-generated documents also contained slightly fewer entity occurrences than the original GS texts, likely because repeated mentions were not always reproduced during rephrasing. This indicates that the current framework was designed primarily to preserve entity forms and boundaries rather than to explicitly control entity frequency. We also note that the generated texts were not evaluated by clinical experts. This study focused on assessing whether persona-driven rephrasings are useful as augmentation data for NER under entity-preservation constraints rather than evaluating the clinical correctness or validity of the generated texts. Therefore, BERTScore and BLEU-4 were used only as descriptive proxy measures of semantic fidelity and lexical variation. Future work should include expert review to assess the factual accuracy and biomedical appropriateness of the generated outputs.

An additional limitation concerns the dependence on GPT-4o, a closed-source proprietary model, for text generation. This raises concerns about accessibility, cost, and reproducibility. Moreover, while the present experiments were conducted in a research setting, the use of external proprietary LLMs may raise privacy, information governance, and potential information leakage concerns in real clinical deployment. Future work should therefore investigate whether open-source or on-premises LLMs can achieve a similar level of fidelity in following XML constraints and preserving entities with minimal alteration. Establishing such alternatives would improve the transparency, accessibility, and practical deployability of persona-driven DA in biomedical settings.

### Conclusion

This study evaluated a persona-driven DA framework for improving biomedical disease NER using document-level rephrasings generated by GPT-4o. By combining XML-constrained prompts with persona-guided rephrasings, the framework produced synthetic data that introduced controlled linguistic variation while largely preserving annotated entities and improved performance over nonaugmented training in several settings.

The analysis further showed that the effectiveness of persona-driven DA depended on both the persona selection strategy and the target corpus. The strongest gains were generally observed when multiple persona-generated variants were combined with the GS data. Most notably, in low-resource experiments on NCBI disease, the all-personas and high-fidelity persona settings outperformed the model trained on 100% of the GS data using only 60% of the training data, suggesting that persona-driven DA can meaningfully reduce annotation requirements without sacrificing performance. Entity-level analysis suggested some additional benefit in RareDis, where augmentation reduced certain confusions between closely related categories.

Overall, these findings support the potential of persona-driven DA as a useful strategy for BioNER in settings with limited annotated data. Further evaluation across other biomedical tasks, datasets, and generation models will help clarify its broader applicability.

## Data Availability

The National Center for Biotechnology Information (NCBI) disease corpus used in this study is publicly available from the National Library of Medicine and National Institutes of Health. The code developed for augmentation and model training, together with the synthetic texts generated from the NCBI disease corpus, is available in our GitHub repository [39]. For the RareDis dataset, access to the original texts is handled by the dataset authors on request. Because our RareDis-based synthetic texts were derived from source materials that are not openly redistributed and may remain close to the original texts, they are not publicly released.

## Appendix

Multimedia Appendix 1 Supplementary dataset statistics, generation-quality metrics, named entity recognition (NER) performance results, entity-preservation analyses, error examples, and low-resource evaluation results for RareDis and National Center for Biotechnology Information (NCBI) disease.

## Abbreviations

BERT: Bidirectional Encoder Representations from Transformers
BioNER: biomedical NER
BLEU: Bilingual Evaluation Understudy
DA: data augmentation
FN: false negative
FP: false positive
GS: gold-standard
LLM: large language model
MUC-6: Message Understanding Conference-6
NCBI: National Center for Biotechnology Information
NER: named entity recognition
NLP: natural language processing
NORD: National Organization for Rare Disorders
RQ: research question
SR: synonym replacement
TP: true positive
