# Supplementary material for: Persona-Driven Data Augmentation for Disease Name Recognition Across Rare and General Disease Corpora: Comparative Evaluation Study
Source: JMIR Med Inform. 2026 Jul 24;14:e87831. doi: 10.2196/87831 (PMC13451868; doi:10.2196/87831)
Supplement: Supplementary file 1 [file medinform_v14i1e87831_app1.docx]

| **Dataset** | **Avg. tokens per doc** | **Avg. entities per doc** | **Rare disease count** | **Disease count** | **Sign count** | **Symptom count** |
| --- | --- | --- | --- | --- | --- | --- |
| RareDis training set | **181.44** | **12.47** | **3610** | **1667** | **3537** | 320 |
| P1 | 163.76 | 12.34 | 3550 | 1650 | 3518 | 322 |
| P2 | 165.94 | 12.32 | 3535 | 1647 | 3525 | 322 |
| P3 | 149.29 | 12.04 | 3390 | 1610 | 3503 | 319 |
| P4 | 168.24 | 12.33 | 3534 | 1646 | 3530 | 320 |
| P5 | 164.04 | 12.19 | 3458 | 1627 | 3522 | 321 |
| P6 | 163.82 | 12.10 | 3415 | 1634 | 3478 | **339** |
| P7 | 165.26 | 12.15 | 3432 | 1634 | 3510 | 323 |
| P8 | 164.98 | 12.09 | 3403 | 1629 | 3501 | 324 |
| P9 | 166.43 | 12.06 | 3422 | 1633 | 3465 | 319 |
| P10 | 170.73 | 12.00 | 3383 | 1630 | 3458 | 323 |

S1: Descriptive statistics of the RareDis corpus. Overall, the RareDis training set shows the highest values for average tokens per document, average entities per document, Rare disease count, Disease count, and Sign count, whereas P6 shows the highest Symptom count.

| **Dataset** | **Avg. tokens per doc** | **Avg. entities per doc** | **Disease count** |
| --- | --- | --- | --- |
| NCBI training set | **215.05** | **8.86** | **5246** |
| P1 | 205.78 | 8.85 | 5237 |
| P2 | 204.44 | 8.83 | 5229 |
| P3 | 195.96 | 8.82 | 5224 |
| P4 | 206.06 | 8.84 | 5233 |
| P5 | 206.28 | 8.83 | 5229 |
| P6 | 203.00 | 8.77 | 5194 |
| P7 | 200.32 | 8.56 | 5067 |
| P8 | 204.50 | 8.80 | 5209 |
| P9 | 205.36 | 8.82 | 5219 |
| P10 | 210.79 | 8.79 | 5201 |

S2: Descriptive statistics of the NCBI disease corpus. The NCBI training set has the highest values for average tokens per document, average entities per document, and Disease count across all compared synthetic datasets.

| **Persona** | **Source** | **BERTScore F1 score** |
| --- | --- | --- |
| P1 | NCBI | 97.71 |
| P1 | RareDis | 96.46 |
| P2 | NCBI | 97.61 |
| P2 | RareDis | 97.02 |
| P3 | NCBI | 97.46 |
| P3 | RareDis | 96.18 |
| P4 | NCBI | 97.58 |
| P4 | RareDis | 97.18 |
| P5 | NCBI | 97.30 |
| P5 | RareDis | 96.77 |
| P6 | NCBI | 96.49 |
| P6 | RareDis | 95.92 |
| P7 | NCBI | 96.73 |
| P7 | RareDis | 96.80 |
| P8 | NCBI | 96.79 |
| P8 | RareDis | 96.00 |
| P9 | NCBI | 96.44 |
| P9 | RareDis | 95.87 |
| P10 | NCBI | 96.37 |
| P10 | RareDis | 95.54 |

S3: BERTScore F1 score values for texts generated by each persona (P1–P10) across the NCBI disease and RareDis datasets.

| **Persona** | **Source** | **BLEU-4 score** |
| --- | --- | --- |
| P1 | NCBI | 65.39 |
| P1 | RareDis | 49.88 |
| P2 | NCBI | 63.26 |
| P2 | RareDis | 55.72 |
| P3 | NCBI | 62.75 |
| P3 | RareDis | 46.78 |
| P4 | NCBI | 64.79 |
| P4 | RareDis | 57.51 |
| P5 | NCBI | 60.70 |
| P5 | RareDis | 52.21 |
| P6 | NCBI | 53.03 |
| P6 | RareDis | 43.76 |
| P7 | NCBI | 59.37 |
| P7 | RareDis | 53.04 |
| P8 | NCBI | 56.29 |
| P8 | RareDis | 44.49 |
| P9 | NCBI | 54.43 |
| P9 | RareDis | 44.23 |
| P10 | NCBI | 53.79 |
| P10 | RareDis | 41.24 |

S4: BLEU-4 score for texts generated by each persona (P1–P10) across the NCBI disease and RareDis datasets.

| **Name** | **Source** | **Dev F1 score mean** | **Dev F1 score standard deviation** |
| --- | --- | --- | --- |
| GS + P1 | NCBI | 87.124 | 0.764 |
| GS + P1 | RareDis | 72.479 | 0.860 |
| GS + P2 | NCBI | **87.257** | 0.420 |
| GS + P2 | RareDis | **73.029** | 0.359 |
| GS + P3 | NCBI | **87.317** | 0.380 |
| GS + P3 | RareDis | 72.909 | 0.516 |
| GS + P4 | NCBI | 87.128 | 0.204 |
| GS + P4 | RareDis | **73.025** | 0.217 |
| GS + P5 | NCBI | 86.946 | 0.683 |
| GS + P5 | RareDis | 72.837 | 0.178 |
| GS + P6 | NCBI | 86.812 | 0.621 |
| GS + P6 | RareDis | 72.928 | 0.407 |
| GS + P7 | NCBI | 86.895 | 0.648 |
| GS + P7 | RareDis | **73.101** | 0.386 |
| GS + P8 | NCBI | **87.135** | 0.431 |
| GS + P8 | RareDis | 72.648 | 0.819 |
| GS + P9 | NCBI | 86.822 | 0.504 |
| GS + P9 | RareDis | 72.919 | 0.097 |
| GS + P10 | NCBI | 86.997 | 0.510 |
| GS + P10 | RareDis | 73.023 | 0.146 |

S5: Mean development-set F1 scores and standard deviations for GS combined with each individual persona on the NCBI disease and RareDis datasets. The top three scores for RareDis are shown in **bold red**, and the top three scores for NCBI disease are shown in **bold blue**.

| **Source** | **Method** | **Precision mean** | **Precision standard deviation** | **Recall mean** | **Recall standard deviation** | **F1 score mean** | **F1 score standard deviation** |
| --- | --- | --- | --- | --- | --- | --- | --- |
| RareDis | Baseline (GS) | 67.86 | 0.56 | 74.94 | 0.37 | 71.22 | 0.45 |
| RareDis | SR | 64.27 (-) | 0.39 | 71.64 (-) | 0.42 | 67.75 (-) | 0.34 |
| RareDis | P1 | 66.2 (-) | 0.57 | 74.4 (-) | 0.60 | 70.06 (-) | 0.58 |
| RareDis | P2 | 67.25 (-) | 0.30 | 74.63 (-) | 0.26 | 70.75 (-) | 0.06 |
| RareDis | P3 | 66.08 (-) | 0.22 | 75.08 (+) | 0.50 | 70.29 (-) | 0.12 |
| RareDis | P4 | 67.41 (-) | 0.31 | 75.16 (+) | 0.10 | 71.07 (-) | 0.21 |
| RareDis | P5 | 66.99 (-) | 0.93 | 74.98 (+) | 0.53 | 70.76 (-) | 0.74 |
| RareDis | P6 | 66.36 (-) | 0.35 | 75.49 (+) | 0.58 | 70.63 (-) | 0.27 |
| RareDis | P7 | 67.12 (-) | **1.2** | 75.14 (+) | **1.06** | 70.9 (-) | **1.14** |
| RareDis | P8 | 66.09 (-) | 0.33 | 74.96 (+) | 0.52 | 70.25 (-) | 0.21 |
| RareDis | P9 | 66.5 (-) | 0.30 | 75.29 (+) | 0.55 | 70.63 (-) | 0.26 |
| RareDis | P10 | 65.82 (-) | 0.39 | 75.98 (+) | 0.36 | 70.53 (-) | 0.13 |
| RareDis | GS + P1 | 69.67 (+) | 0.97 | 75.9 (+) | 0.56 | 72.65 (+) | 0.79 |
| RareDis | GS + P2 | 69.74 (+) | 0.72 | 75.37 (+) | 0.48 | 72.44 (+) | 0.52 |
| RareDis | GS + P3 | 69.15 (+) | 0.19 | 75.56 (+) | 0.26 | 72.21 (+) | 0.10 |
| RareDis | GS + P4 | 69.19 (+) | 0.66 | 76.1 (+) | 0.30 | 72.48 (+) | 0.29 |
| RareDis | GS + P5 | 69.34 (+) | 0.11 | 75.83 (+) | 0.20 | 72.44 (+) | 0.06 |
| RareDis | GS + P6 | 69.96 (+) | 0.54 | 75.53 (+) | 0.44 | 72.64 (+) | 0.37 |
| RareDis | GS + P7 | 69.53 (+) | 0.41 | 75.94 (+) | 0.04 | 72.59 (+) | 0.24 |
| RareDis | GS + P8 | 69.64 (+) | 0.29 | 75.57 (+) | 0.66 | 72.48 (+) | 0.22 |
| RareDis | GS + P9 | 69.2 (+) | 0.67 | 75.21 (+) | 0.72 | 72.08 (+) | 0.40 |
| RareDis | GS + P10 | 69.26 (+) | 0.52 | 75.86 (+) | 0.39 | 72.41 (+) | 0.46 |
| RareDis | GS + SR | 68.73 (+) | 0.28 | 75.73 (+) | 0.56 | 72.06 (+) | 0.41 |
| RareDis | GS + High-fidelity personas | 70.26 (+) | 0.36 | 74.9 (-) | 0.54 | 72.5 (+) | 0.32 |
| RareDis | GS + Balanced-fidelity personas | 70.24 (+) | 0.44 | **76.18 (+)** | 0.99 | 73.09 (+) | 0.33 |
| RareDis | GS + Low-fidelity personas | 71.31 (+) | 0.43 | 75.52 (+) | 0.10 | **73.35 (+)** | 0.19 |
| RareDis | GS + Top-3 performing personas | 70.57 (+) | 0.48 | 75.7 (+) | 0.79 | 73.05 (+) | 0.56 |
| RareDis | GS + All personas | **71.56 (+)** | 1.17 | 75.16 (+) | 0.43 | 73.31 (+) | 0.73 |

S6: Mean precision, recall, and F1 scores (with standard deviations) for the RareDis evaluation. Values in parentheses indicate change relative to the gold-standard baseline (Baseline [GS]), where (+) denotes an increase and (−) denotes a decrease.

| **Source** | **Method** | **Precision mean** | **Precision standard deviation** | **Recall mean** | **Recall standard deviation** | **F1 score mean** | **F1 score standard deviation** |
| --- | --- | --- | --- | --- | --- | --- | --- |
| NCBI | Baseline (GS) | 85.52 | 0.67 | 90.25 | 0.38 | 87.82 | 0.18 |
| NCBI | SR | 80.0 (-) | **1.45** | 87.5 (-) | 0.69 | 83.58 (-) | 0.93 |
| NCBI | P1 | 82.46 (-) | 0.59 | 89.97 (-) | 0.74 | 86.05 (-) | 0.59 |
| NCBI | P2 | 83.11 (-) | 0.94 | 90.47 (+) | 0.49 | 86.63 (-) | 0.40 |
| NCBI | P3 | 82.99 (-) | 0.38 | 90.43 (+) | 0.34 | 86.55 (-) | 0.05 |
| NCBI | P4 | 82.05 (-) | 0.72 | 90.01 (-) | **1.12** | 85.84 (-) | 0.27 |
| NCBI | P5 | 82.49 (-) | 0.81 | 90.01 (-) | 0.75 | 86.09 (-) | 0.75 |
| NCBI | P6 | 81.34 (-) | 0.56 | 89.58 (-) | 0.22 | 85.26 (-) | 0.37 |
| NCBI | P7 | 82.14 (-) | 1.34 | 89.72 (-) | 0.64 | 85.76 (-) | **1.01** |
| NCBI | P8 | 82.22 (-) | 0.18 | 89.62 (-) | 0.56 | 85.76 (-) | 0.27 |
| NCBI | P9 | 81.32 (-) | 1.10 | **90.61 (+)** | 0.75 | 85.71 (-) | 0.32 |
| NCBI | P10 | 81.42 (-) | 0.43 | 89.58 (-) | 0.31 | 85.31 (-) | 0.32 |
| NCBI | GS + P1 | 86.92 (+) | 0.38 | 90.32 (+) | 0.52 | 88.59 (+) | 0.38 |
| NCBI | GS + P2 | 86.49 (+) | 0.38 | 89.72 (-) | 0.56 | 88.08 (+) | 0.43 |
| NCBI | GS + P3 | 86.81 (+) | 0.75 | 90.15 (-) | 0.28 | 88.45 (+) | 0.51 |
| NCBI | GS + P4 | 87.13 (+) | 0.42 | 90.08 (-) | 0.62 | 88.58 (+) | 0.42 |
| NCBI | GS + P5 | 87.47 (+) | 0.19 | 90.25 (+) | 0.11 | 88.84 (+) | 0.15 |
| NCBI | GS + P6 | 86.71 (+) | 1.31 | 90.47 (+) | 0.55 | 88.55 (+) | 0.94 |
| NCBI | GS + P7 | 87.57 (+) | 0.58 | 90.5 (+) | 0.16 | 89.01 (+) | 0.22 |
| NCBI | GS + P8 | 86.91 (+) | 0.48 | 89.97 (-) | 0.69 | 88.41 (+) | 0.23 |
| NCBI | GS + P9 | 86.59 (+) | 0.52 | 89.62 (-) | 0.73 | 88.08 (+) | 0.56 |
| NCBI | GS + P10 | 86.51 (+) | 0.80 | 89.87 (-) | 0.49 | 88.15 (+) | 0.65 |
| NCBI | GS + SR | 87.36 (+) | 0.23 | 90.04 (-) | 0.28 | 88.68 (+) | 0.18 |
| NCBI | GS + High-fidelity personas | 88.16 (+) | 0.26 | 90.47 (+) | 0.32 | 89.3 (+) | 0.22 |
| NCBI | GS + Balanced-fidelity personas | 87.43 (+) | 0.71 | 90.57 (+) | 0.80 | 88.97 (+) | 0.63 |
| NCBI | GS + Low-fidelity personas | 87.83 (+) | 0.53 | 90.18 (-) | 0.53 | 88.99 (+) | 0.51 |
| NCBI | GS + Top-3 performing personas | 88.14 (+) | 0.56 | 90.25 (+) | 1.04 | 89.18 (+) | 0.79 |
| NCBI | GS + All personas | **88.58 (+)** | 0.71 | 90.08 (-) | 0.24 | **89.32 (+)** | 0.26 |

S7: Mean precision, recall, and F1 scores (with standard deviations) for the NCBI evaluation. Values in parentheses indicate change relative to the gold-standard baseline (Baseline [GS]), where (+) denotes an increase and (−) denotes a decrease.

| **Source** | **Method** | **Disease** | **Disease standard deviation** | **Rare disease** | **Rare disease standard deviation** | **Sign** | **Sign standard deviation** | **Symptom** | **Symptom standard deviation** |
| --- | --- | --- | --- | --- | --- | --- | --- | --- | --- |
| RareDis | Baseline (GS) | 63.17 | **1.39** | 86.56 | 0.14 | 59.26 | 1.02 | 52.93 | 1.36 |
| RareDis | SR | 55.87 (-) | 0.40 | 84.67 (-) | 0.28 | 55.81 (-) | 0.61 | 48.41 (-) | 1.37 |
| RareDis | P1 | 61.86 (-) | 1.00 | 86.35 (-) | 0.24 | 57.34 (-) | 0.78 | 52.72 (-) | 0.95 |
| RareDis | P2 | 61.9 (-) | 0.73 | 86.42 (-) | 0.25 | 58.94 (-) | 1.30 | 51.55 (-) | 3.51 |
| RareDis | P3 | 62.09 (-) | 1.04 | 86.69 (+) | 0.50 | 57.41 (-) | 0.59 | 54.6 (+) | 0.38 |
| RareDis | P4 | 61.08 (-) | 0.59 | 86.59 (+) | 0.22 | 59.8 (+) | 0.49 | 54.29 (+) | 0.92 |
| RareDis | P5 | 61.56 (-) | 0.56 | 86.65 (+) | 0.31 | 58.66 (-) | **1.45** | 55.21 (+) | 0.66 |
| RareDis | P6 | 60.98 (-) | 0.93 | 86.89 (+) | 0.27 | 58.4 (-) | 0.45 | 59.26 (+) | 5.97 |
| RareDis | P7 | 61.86 (-) | 1.19 | 86.66 (+) | 0.34 | 58.94 (-) | 1.30 | 53.87 (+) | 4.80 |
| RareDis | P8 | 60.44 (-) | 1.03 | 87.12 (+) | 0.34 | 57.47 (-) | 0.72 | 56.58 (+) | 3.17 |
| RareDis | P9 | 61.03 (-) | 1.31 | 87.04 (+) | 0.24 | 58.28 (-) | 0.74 | 58.46 (+) | 5.70 |
| RareDis | P10 | 61.85 (-) | 0.73 | 86.68 (+) | 0.47 | 58.25 (-) | 0.24 | 57.84 (+) | 0.56 |
| RareDis | GS + P1 | 65.14 (+) | 0.77 | 87.05 (+) | 0.72 | 60.69 (+) | 1.23 | 64.02 (+) | 0.30 |
| RareDis | GS + P2 | 64.25 (+) | 0.34 | 87.05 (+) | 0.52 | 60.44 (+) | 0.20 | 65.11 (+) | 0.58 |
| RareDis | GS + P3 | 64.39 (+) | 0.60 | 86.82 (+) | 0.28 | 60.34 (+) | 0.38 | 63.03 (+) | 0.89 |
| RareDis | GS + P4 | 64.52 (+) | 0.32 | 87.18 (+) | 0.52 | 60.67 (+) | 0.58 | 64.59 (+) | 2.94 |
| RareDis | GS + P5 | 64.03 (+) | 0.18 | 86.99 (+) | 0.16 | 60.97 (+) | 0.24 | 63.86 (+) | 2.67 |
| RareDis | GS + P6 | 64.14 (+) | 0.19 | 87.55 (+) | **0.95** | 60.21 (+) | 0.66 | 65.91 (+) | 1.76 |
| RareDis | GS + P7 | 64.7 (+) | 0.65 | 86.99 (+) | 0.27 | 60.95 (+) | 0.10 | 65.11 (+) | 2.59 |
| RareDis | GS + P8 | 63.99 (+) | 0.50 | 87.2 (+) | 0.10 | 60.34 (+) | 0.57 | 65.92 (+) | 0.63 |
| RareDis | GS + P9 | 64.06 (+) | 0.26 | 86.74 (+) | 0.16 | 60.09 (+) | 0.93 | 65.13 (+) | 1.48 |
| RareDis | GS + P10 | 64.29 (+) | 0.26 | 87.12 (+) | 0.23 | 60.38 (+) | 0.78 | 66.1 (+) | 1.85 |
| RareDis | GS + SR | 63.24 (+) | 0.65 | 87.06 (+) | 0.17 | 60.35 (+) | 0.71 | 62.15 (+) | 2.11 |
| RareDis | GS + High-fidelity personas | 63.97 (+) | 0.67 | 86.69 (+) | 0.53 | 60.93 (+) | 0.57 | 67.26 (+) | **6.19** |
| RareDis | GS + Balanced-fidelity personas | 65.42 (+) | 0.39 | 87.5 (+) | 0.07 | 61.16 (+) | 0.68 | 66.5 (+) | 2.30 |
| RareDis | GS + Low-fidelity personas | 65.64 (+) | 0.23 | 87.06 (+) | 0.33 | **61.95 (+)** | 0.28 | **68.47 (+)** | 2.79 |
| RareDis | GS + Top-3 performing personas | **65.66 (+)** | 1.00 | 87.53 (+) | 0.24 | 60.9 (+) | 0.76 | 65.7 (+) | 3.36 |
| RareDis | GS + All personas | 64.81 (+) | 0.07 | **87.69 (+)** | 0.65 | 61.46 (+) | 0.45 | 68.31 (+) | 1.94 |

S8: Per-entity mean F1 scores and standard deviations for the RareDis test set under no augmentation, SR-based augmentation, and persona-based augmentation settings. (+) indicates an increase relative to the gold-standard baseline (Baseline [GS]), and (−) indicates a decrease. The highest scores were obtained by Top-3 performing personas for Disease, All personas for Rare disease, and Low-fidelity personas for both Sign and Symptom.

| **Source** | **Method** | **Disease** | **Disease standard deviation** |
| --- | --- | --- | --- |
| NCBI | Baseline (GS) | 87.82 | 0.18 |
| NCBI | SR | 83.58 (-) | 0.93 |
| NCBI | P1 | 86.05 (-) | 0.59 |
| NCBI | P2 | 86.63 (-) | 0.40 |
| NCBI | P3 | 86.55 (-) | 0.05 |
| NCBI | P4 | 85.84 (-) | 0.27 |
| NCBI | P5 | 86.09 (-) | 0.75 |
| NCBI | P6 | 85.26 (-) | 0.37 |
| NCBI | P7 | 85.76 (-) | **1.01** |
| NCBI | P8 | 85.76 (-) | 0.27 |
| NCBI | P9 | 85.71 (-) | 0.32 |
| NCBI | P10 | 85.31 (-) | 0.32 |
| NCBI | GS + P1 | 88.59 (+) | 0.38 |
| NCBI | GS + P2 | 88.08 (+) | 0.43 |
| NCBI | GS + P3 | 88.45 (+) | 0.51 |
| NCBI | GS + P4 | 88.58 (+) | 0.42 |
| NCBI | GS + P5 | 88.84 (+) | 0.15 |
| NCBI | GS + P6 | 88.55 (+) | 0.94 |
| NCBI | GS + P7 | 89.01 (+) | 0.22 |
| NCBI | GS + P8 | 88.41 (+) | 0.23 |
| NCBI | GS + P9 | 88.08 (+) | 0.56 |
| NCBI | GS + P10 | 88.15 (+) | 0.65 |
| NCBI | GS + SR | 88.68 (+) | 0.18 |
| NCBI | GS + High-fidelity personas | 89.3 (+) | 0.22 |
| NCBI | GS + Balanced-fidelity personas | 88.97 (+) | 0.63 |
| NCBI | GS + Low-fidelity personas | 88.99 (+) | 0.51 |
| NCBI | GS + Top-3 performing personas | 89.18 (+) | 0.79 |
| NCBI | GS + All personas | **89.32 (+)** | 0.26 |

S9: Per-entity mean F1 scores and standard deviations for the NCBI disease test set under no augmentation, SR-based augmentation, and persona-based augmentation settings. (+) indicates an increase relative to the gold-standard baseline (Baseline [GS]), and (−) indicates a decrease. The best score was achieved by combining GS with all personas for the Disease entity.

| **Source** | **Sentence** | **Baseline (GS) token predictions** | **Best augmented method token predictions** |
| --- | --- | --- | --- |
| RareDis test set | …(<raredisease>CPEO</raredisease>); abnormal accumulation of colored … | CPEO (O) | CPEO (B-RAREDISEASE) |
| RareDis test set | … <raredisease>Pneumocystis pneumonia</raredisease> is characterized … | Pneumocystis (B-DISEASE);  pneumonia (I-DISEASE) | Pneumocystis (B-DISEASE);  pneumonia (I-DISEASE) |
| RareDis test set | … When <raredisease>tetralogy of Fallot</raredisease> … | tetralogy (B-RAREDISEASE);  of (I-RAREDISEASE);  Fallot (I-RAREDISEASE) | tetralogy (B-DISEASE);  of (I-DISEASE);  Fallot (I-DISEASE) |
| RareDis test set | … not have symptoms (<symptom>asymptomatic</symptom>) … | asymptomatic (B-SIGN) | asymptomatic (B-SYMPTOM) |
| RareDis test set | … indicates <symptom>pain</symptom>. … | pain (B-SYMPTOM) | pain (O) |
| RareDis test set | causing <symptom>increased appetite</symptom> … | increased (B-SIGN);  appetite (I-SIGN) | increased (B-SYMPTOM);  appetite (I-SIGN) |
| NCBI test set | … and <disease>mild hyperphenylalaninemia</disease> … | mild (B-DISEASE);  hyperphenylalaninemia (B-DISEASE) | mild (B-DISEASE);  hyperphenylalaninemia (I-DISEASE) |
| NCBI test set | developed <disease>bilateral retinoblastoma</disease>. | bilateral (B-DISEASE);  retinoblastoma(B-DISEASE) | bilateral (O);  retinoblastoma (B-DISEASE) |
| NCBI test set | … resulting in <disease>maternal UPD 15</disease> … | maternal (O);  UPD (I-DISEASE);  15 (I-DISEASE) | maternal (O);  UPD (I-DISEASE);  15 (I-DISEASE) |

S10: Representative error analysis examples from the RareDis and NCBI disease test sets, comparing token-level predictions from the GS baseline and the best-performing augmented method.

| **Persona** | **Source** | **GS total entity count** | **Modified entity count** | **Modified entity percentage** |
| --- | --- | --- | --- | --- |
| P1 | RareDis | 9134 | 98 | 1.07 |
| P2 | RareDis | 9134 | 84 | 0.92 |
| P3 | RareDis | 9134 | 91 | 1.00 |
| P4 | RareDis | 9134 | 72 | 0.79 |
| P5 | RareDis | 9134 | 83 | 0.91 |
| P6 | RareDis | 9134 | 146 | 1.60 |
| P7 | RareDis | 9134 | 109 | 1.19 |
| P8 | RareDis | 9134 | 92 | 1.01 |
| P9 | RareDis | 9134 | 126 | 1.38 |
| P10 | RareDis | 9134 | 134 | 1.47 |

S11: Entity modification analysis for RareDis persona-generated variants. Out of 9,134 total GS entities, the proportion of modified entities per persona remained at or below 2% (0.79–1.60), with P6 showing the highest absolute count (146 modified entities). This indicates that XML-constrained prompting largely preserved annotated entity spans across all personas.

| **Persona** | **Source** | **GS total entity count** | **Modified entity count** | **Modified entity percentage** |
| --- | --- | --- | --- | --- |
| P1 | NCBI | 5246 | 11 | 0.21 |
| P2 | NCBI | 5246 | 11 | 0.21 |
| P3 | NCBI | 5246 | 12 | 0.23 |
| P4 | NCBI | 5246 | 12 | 0.23 |
| P5 | NCBI | 5246 | 13 | 0.25 |
| P6 | NCBI | 5246 | 16 | 0.30 |
| P7 | NCBI | 5246 | 16 | 0.30 |
| P8 | NCBI | 5246 | 12 | 0.23 |
| P9 | NCBI | 5246 | 19 | 0.36 |
| P10 | NCBI | 5246 | 22 | 0.42 |

S12: Entity modification analysis for NCBI disease persona-generated variants. Out of 5,246 total GS entities, all personas showed a modification rate between 0.2% and 0.4%, with absolute counts ranging from 11 (P1, P2) to 22 (P10). This demonstrates near-perfect entity preservation across all personas in the NCBI disease setting.

| **Batch DA Method** | **Source** | **Training data percentage** | **F1 score mean** | **F1 score standard deviation** |
| --- | --- | --- | --- | --- |
| GS | RareDis | 20 | 61.59 | 1.07 |
| GS | RareDis | 40 | 67.19 | 0.12 |
| GS | RareDis | 60 | 68.79 | 0.19 |
| GS | RareDis | 80 | 69.81 | 0.53 |
| GS | RareDis | 100 | 71.22 | 0.45 |
| GS + SR | RareDis | 20 | 61.63 | 0.46 |
| GS + SR | RareDis | 40 | 65.32 | 0.76 |
| GS + SR | RareDis | 60 | 67.20 | 0.48 |
| GS + SR | RareDis | 80 | 67.9 | 0.65 |
| GS + SR | RareDis | 100 | 72.06 | 0.41 |
| GS + Low-fidelity personas | RareDis | 20 | 63.74 | 0.48 |
| GS + Low-fidelity personas | RareDis | 40 | 66.91 | 0.14 |
| GS + Low-fidelity personas | RareDis | 60 | 68.77 | 0.37 |
| GS + Low-fidelity personas | RareDis | 80 | 69.10 | 0.29 |
| GS + Low-fidelity personas | RareDis | 100 | 73.35 | 0.19 |
| GS + All personas | RareDis | 20 | 64.42 | 0.59 |
| GS + All personas | RareDis | 40 | 67.81 | 0.56 |
| GS + All personas | RareDis | 60 | 68.94 | 0.11 |
| GS + All personas | RareDis | 80 | 69.96 | 0.74 |
| GS + All personas | RareDis | 100 | 73.31 | 0.73 |

S13: Performance comparison of different DA strategies on the RareDis dataset under varying training-data percentages.

| **Batch DA Method** | **Source** | **Training data percentage** | **F1 score mean** | **F1 score standard deviation** |
| --- | --- | --- | --- | --- |
| GS | NCBI | 20 | 80.99 | 0.54 |
| GS | NCBI | 40 | 84.21 | 2.37 |
| GS | NCBI | 60 | 86.14 | 1.09 |
| GS | NCBI | 80 | 86.86 | 0.43 |
| GS | NCBI | 100 | 87.82 | 0.18 |
| GS + SR | NCBI | 20 | 82.93 | 1.83 |
| GS + SR | NCBI | 40 | 85.51 | 1.47 |
| GS + SR | NCBI | 60 | 86.63 | 1.88 |
| GS + SR | NCBI | 80 | 87.08 | 0.73 |
| GS + SR | NCBI | 100 | 88.68 | 0.18 |
| GS + High-fidelity personas | NCBI | 20 | 84.06 | 1.11 |
| GS + High-fidelity personas | NCBI | 40 | 85.88 | 1.21 |
| GS + High-fidelity personas | NCBI | 60 | 87.88 | 0.55 |
| GS + High-fidelity personas | NCBI | 80 | 88.08 | 0.47 |
| GS + High-fidelity personas | NCBI | 100 | 89.3 | 0.22 |
| GS + All personas | NCBI | 20 | 84.22 | 0.95 |
| GS + All personas | NCBI | 40 | 87.07 | 1.37 |
| GS + All personas | NCBI | 60 | 88.48 | 0.21 |
| GS + All personas | NCBI | 80 | 88.63 | 0.64 |
| GS + All personas | NCBI | 100 | 89.32 | 0.26 |

S14: Performance comparison of different DA strategies on the NCBI disease dataset under varying training-data percentages.
